# Self-assembled monolayers of mesoionic triazolylidene dimers on Au(111)

**DOI:** 10.1039/d5nr02802g

**Published:** 2025-10-24

**Authors:** Iris Berg, Luca Schio, Masoumeh Alihosseini, Justus Reitz, Elena Molteni, Shuangying Ma, Carolina Gutiérrez Bolaños, Andrea Goldoni, Cesare Grazioli, Max M. Hansmann, Guido Fratesi, Luca Floreano, Elad Gross

**Affiliations:** a Institute of Chemistry and The Center for Nanoscience and Nanotechnology, The Hebrew University Jerusalem 91904 Israel elad.gross@mail.huji.ac.il; b CNR-IOM, Istituto Officina dei Materiali Basovizza SS-14 Km 163.5 Trieste 34149 Italy floreano@iom.cnr.it; c Dipartimento di Fisica “Aldo Pontremoli” Università degli Studi di Milano Via Celoria 16 20133 Milano Italy guido.fratesi@unimi.it; d Technische Universität Dortmund, Fakultät für Chemie und Chemische Biologie Otto-Hahn-Str. 6 44227 Dortmund Germany; e Elettra-Sincrotrone Trieste S.C.p.A Basovizza SS-14 Km 163.5 Trieste 34149 Italy

## Abstract

Mesoionic carbenes (MICs) hold great promise as surface ligands, due to their electronic properties and charge distribution, yet their self-assembly rules remain essentially unexplored. Here we combine synchrotron X-ray photoelectron and absorption spectroscopies, scanning-tunnelling microscopy, and density-functional theory to map, atom by atom, the self-assembly of 1,2,3-triazolylidene MICs on Au(111). We discover that the molecules adsorb flat, pair *via* a shared Au adatom, and form two highly ordered phases whose lattice constants differ by ∼5%. The resulting monolayers reach high coverages (1.4–1.5 molecules per nm^2^) while retaining long-range order. X-ray photoelectron spectroscopy and near-edge X-ray absorption fine structure reveal pronounced charge transfer into the metal and a molecule–adatom–molecule motif that lifts the Au adatom by ∼0.8 Å, in excellent agreement with theory. The molecules exhibit thermal stability up to 200 °C, after which they desorb from the surface without detectable decomposition. By elucidating how the mesoionic electronic structure directs adatom extraction, dimer formation, and high-density packing, this work establishes MICs as a versatile platform for stable, strongly coupled organic-metal interfaces.

## Abstract 2

Atomic-scale elucidation of mesoionic carbene monolayers has been achieved, revealing a molecule–adatom–molecule architecture on Au(111).

## Graphical abstract

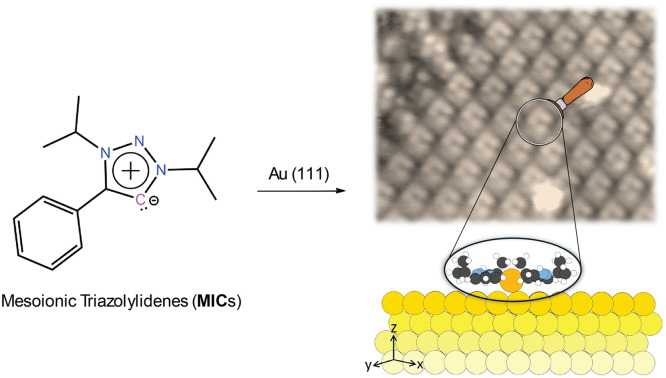

## Introduction

N-heterocyclic carbenes (NHCs) have drawn considerable attention as a robust and versatile platform for surface modification.^1–14^ Their ability to form stable self-assembled monolayers on metal and non-metal surfaces marked them as a viable alternative to commonly used monolayers such as thiols and phosphines, which demonstrated limited stability under ambient conditions.^15^ The extensive research led to multiple applications of NHC based monolayers, such as sensors, co-catalysts, molecular electronics, surface passivation, and surface patterning.^10,14,16–29^

**Scheme 1:**
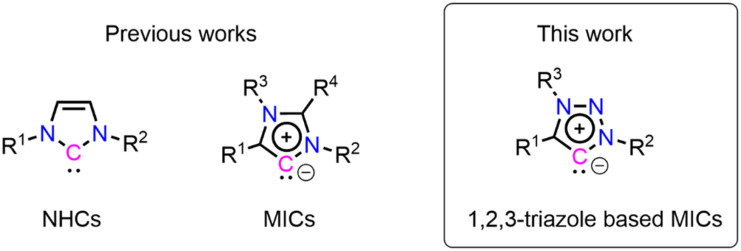
General structures of N-heterocyclic carbene (NHC), Mesoionic carbenes (MIC), and 1,2,3-triazolylidene.

Most of the current literature has focused on neutral-carbenes.^10,14,30–32^ Mesoionic carbenes (MICs),^33–38^ in contrast, exhibit a more pronounced charge separation than NHCs (Scheme 1) and can serve as stronger surface ligandsdue to their stronger σ-donor properties. MICs display a lower Tolman electronic parameter (ca. 2032–2040 cm^−1^) compared to NHCs (*ca.* 2050–2055 cm^−1^ for imidazolidine and imidazol-based NHCs), indicating their overall stronger donor properties.^39^ These characteristics make MICs particularly well-suited as surface ligands.

In a recent study, it was demonstrated that mesoionic carbenes derived from imidazo[1,2-*a*]pyridines can be self-assembled on gold surfaces and are resistant to ligand exchange with NHCs.^40^ It was also shown that triazole-based MIC monolayers on Au films feature higher thermal stabilities, lower work functions, and increased hydrophilicity of the surface, in comparison to NHC-based monolayers.^41,42^ These studies provide initial indications about the crucial impact of the charge state on the self-assembly of MICs. However, molecular level analysis of the self-assembly pattern, the long-range order of MICs on surfaces, and the ways by which these properties are influenced by the unique electronic properties of MICs, have not been elucidated yet.

In this study, we present a thorough experimental and theoretical analysis of the self-assembly of MICs on Au(111), while using the 1,2,3-triazole heterocycle (1,2,3-triazol-5-ylidenes)^33,43–45^ as a model system for MICs (Scheme 1), due to its synthetic accessibility *via* “click” azide–alkyne chemistry. The Tolman electronic parameter of 1,2,3-triazol-5-ylidenes lie around 2039 cm^−1^, which positions them as very strong donor ligands exceeding the donor properties of classical imidazolylidene based carbenes. The self-assembly properties of MIC on Au(111) were analyzed by conducting X-ray photoelectron spectroscopy (XPS), near edge X-ray absorption fine structure (NEXAFS), and scanning tunneling microscopy (STM) measurements, accompanied by density functional theory (DFT) calculations. The results demonstrate that triazole-based MICs self-assemble on gold to form a monolayer with a flat-lying adsorption configuration and a long-range order, characterized by a molecule–adatom–molecule motif. The monolayers exhibit high surface density and notable thermal stability, remaining on the surface to a large extent up to 200 °C before desorbing from the surface without decomposition, demonstrating their molecular robustness.

## Results and discussion

**Scheme 2:**
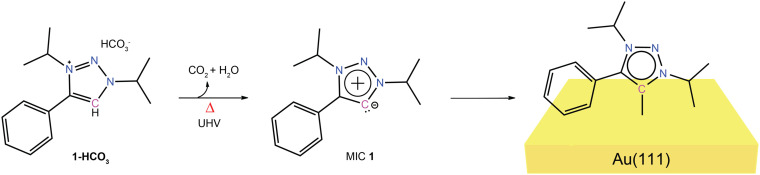
Molecular structures of hydrogen carbonate salt 1-HCO_3_ and triazole-based MIC 1 studied in this work.

Triazole-MIC 1 was self-assembled under ultra-high vacuum (UHV) conditions on Au(111) using the corresponding bicarbonate salt 1-HCO_3_ as a precursor, featuring *N*-isopropyl (iPr) substitution at both 1,3-N-atoms (Scheme 2, see Fig. S1–S6 and SI for experimental details). We have recently observed that MIC 1 shows high stability in solution phase.^46^ Thus, this molecule was preferred over MIC with larger *N*-aryl substituents,^33^ which might decompose upon sublimation. The bicarbonate salt was conveniently prepared by anion exchange from the triflate salt (see SI for experimental details).^2^ The desired triazole-based MIC 1 was generated *in situ* by deprotonation of 1-HCO_3_, which takes place under mild annealing (358 K) in UHV conditions. The hydrogen carbonate salt was chosen as a precursor because it thermally deprotonates *in vacuo* to give MIC 1 with only CO_2_ and H_2_O as volatile by-products, thus eliminating the need for an addition of external base and minimizing the concentration of counterion residues on the surface. The application of the bicarbonate precursor is a standard route for vacuum deposition of carbenes on Au(111).^2,16,30,47–51^

**Fig. 1:**
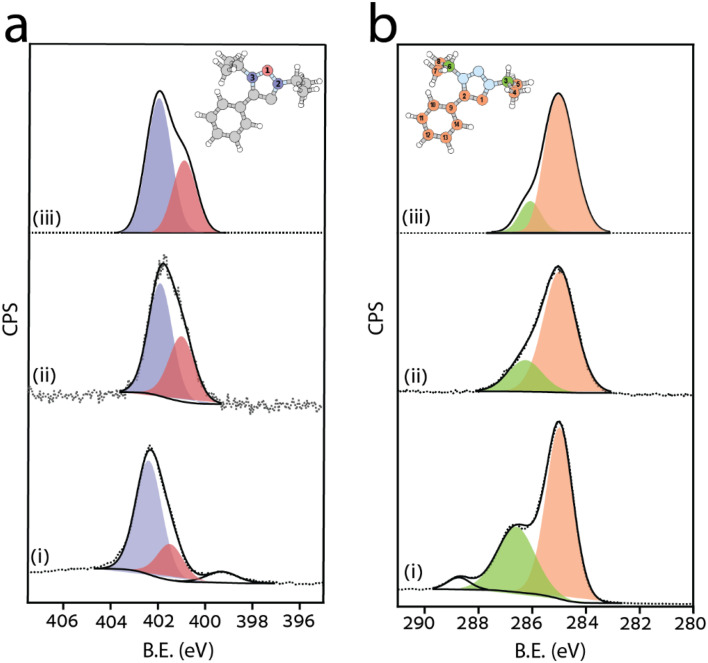
(a) N 1s and (b) C 1s XP-spectra of (i) 1-HCO_3_ precursor, (ii) as-deposited MIC 1 and (iii) simulated XP-spectra for gas-phase MIC 1. The dotted lines show the acquired data, and the solid lines show the Gaussian fitting. The center of the simulated data is aligned to the experimental one. The insets display the molecular structure with numbered nitrogen and carbon atoms; atomic species are colored according to the corresponding fitting components.

The monolayer phase ordering, the adsorption geometry, and its thermal stability were measured by synchrotron-based XPS and linearly polarized NEXAFS, performed at the ALOISA beamline of the Elettra Synchrotron (Trieste, Italy).^52^ As a reference, XPS measurements of the precursor salt in a multilayer film (prepared *ex situ* by drop casting) were conducted. The N 1s photoemission spectra of the precursor (Fig. 1a, spectrum i) show two main components that can be fitted to Gaussian profiles peaked at binding energy (BE) of 402.4 and 401.4 eV. The area ratio between the Gaussians was *ca.* 2 : 1, implying that the main component is associated with the N2 and N3 nitrogen atoms^53,54^ (Fig. 1a, inset), whereas the component at lower binding energy is assigned to N1 species, *i.e.* the central nitrogen atom in the triazole backbone. This assignment is in full agreement with DFT simulations for the gas phase molecule (Fig. 1a, spectrum (iii)), where the N2 and N3 nitrogen atom peaks are shifted towards higher binding energies with respect to that of the N1 species. An additional minor component was observed in the precursor's spectrum at BE = 399.3 eV due to residual contamination during the precursor film preparation and its exposure to air.

Upon sublimation under UHV conditions, 1-HCO_3_ deprotonates for the formation of MIC 1 that is anchored to the surface. The corresponding N 1s XP spectrum showed a profile similar to the precursor one, well fitted by two Gaussians positioned at 401.9 eV and 400.9 eV (Fig. 1a, spectrum ii). The monolayer signal was shifted to lower binding energies by 0.5 eV with respect to the precursor. Such a core level shift (CLS) is consistent with a final state effect due to the surface screening by Fermi metal electrons, possibly incremented by electron back-donation from the metal to the electron withdrawing triazole ring. For comparison, a SAM of triazole-based MIC 1 was also prepared on a gold film by the addition of an external organic base. This alternative route to SAM preparation yields the same molecular species, as observed by analysis of the N 1s XP spectrum (Fig. S7).

The C 1s XP spectrum of the precursor (Fig. 1b, spectrum (i)) was comprised of three Gaussians, positioned at 285.0, 286.6, and 288.7 eV and correlated to C–C, C–N, and C=O, respectively.^55,56^ The high binding energy component in the C 1s XP spectrum vanished following deprotonation and surface deposition of MIC 1 on Au (111) (spectrum ii), consistent with the loss of CO_2_ and water during active carbene formation. The DFT-simulated XP spectrum for an isolated MIC 1 molecule mostly puts in evidence the large CLS of the alkyl carbons (C3 and C6) that are bound to nitrogen with respect to all the other carbon atoms (Fig. 1b, spectrum (iii)). Such a simple regrouping of the carbon atom contributions into two main components of C 1s may yield an effective fitting of the experimental spectrum (Fig. 1b, spectrum (ii)). However, high resolution XPS from the adsorbed species clearly shows a multicomponent fine structure of the main C 1s peak at 285–286 eV. The splitting among the carbon components may be partly attributed to different screening from the metal Fermi electrons due to different atomic distance from the surface, as well as to chemical interaction of the ligand carbon atom with the substrate.

**Fig. 2:**
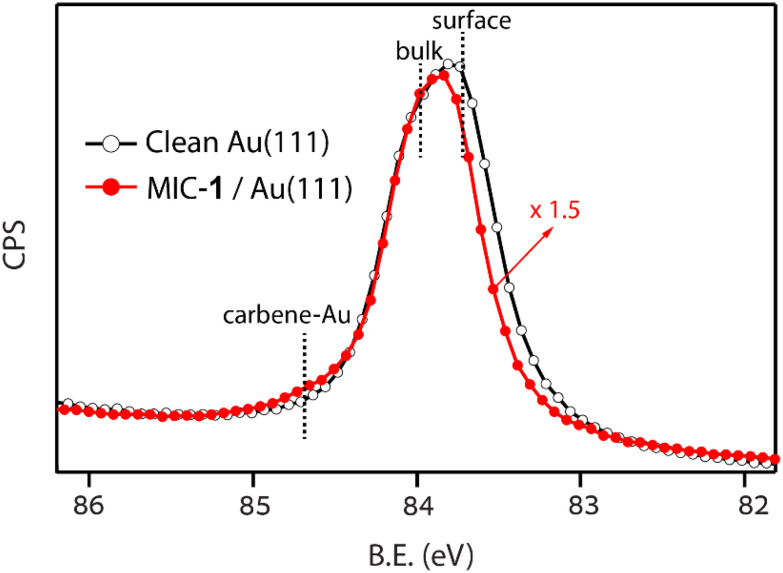
Photoemission spectra of the Au 4f_7/2_ peak measured at a photon energy of 160 eV (Δ*E* = 110 meV) on the pristine Au(111) surface (black line with open markers) and after the deposition of a saturated SAM of MIC-1 (red line with filled markers).

For a better insight into the nature of the interaction of MIC 1 with the surface, we measured the XPS of Au 4f at a photon energy of 160 eV, where the surface component for the clean Au(111) surface is clearly resolved and shifted by −0.3 eV to lower BE with respect to the bulk signal (black-colored spectrum, Fig. 2).^57^ The changes in the Au 4f_7/2_ peak after SAM deposition is clearly shown (red colored spectrum, Fig. 2), where a new small component is observed at ∼0.6 eV higher BE with respect to the bulk Au component. The surface component shows a clear shift to higher BE by ∼0.1 eV.

These spectral changes are very similar to those originally reported for a methylthiol (CH_3_S) SAM on Au(111),^57^ and more recently for NHC/Au(111),^58^ yielding a CLS of 0.4 and 0.9 eV, respectively. In both cases, the new component is associated with the extraction of Au atoms by the simultaneous linking to two thiols^59^ or two NHCs.^58^ As we shall see when discussing the computed adsorption structures, the Au atoms that are coordinated to MIC 1 are lifted further away from the surface by nearly ∼0.8 Å with respect to an isolated Au adatom (lying ∼2.0 Å above the surface plane). Thus, the measured binding energy of the adatom component is the result of a combination of chemical shift (depending on the nature of the ligand) and of the screening from the metal (depending on the distance from the surface).

**Fig. 3:**
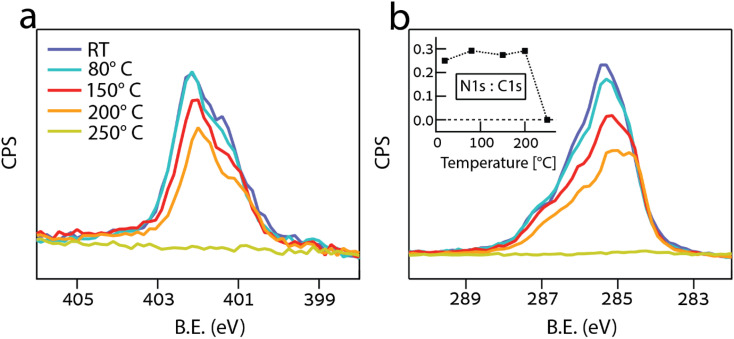
(a) N 1s and (b) C 1s XP-spectra of as-deposited MIC 1 monolayer as a function of increasing annealing temperature. The spectra have been calibrated and renormalized to the Fermi edge. The corresponding intensity ratio between the N 1s and C 1s spectra is shown in the inset of (b).

The thermal stability of the triazole-MIC monolayer was assessed by measuring the XPS signal after annealing to increasingly higher temperatures. The corresponding N 1s and C 1s photoemission spectra of the MIC 1 SAM are presented in Fig. 3. Apart from a ∼25% intensity decrease between 150 and 200 °C, most of the molecules remain on the surface up to 200 °C. In between 150 and 200 °C, we observe a small CLS to lower binding energy of both the N 1s and C 1s components, as well as a small change of the C 1s line shape. We attribute this small spectral evolution to a change of the adsorption site (the CLS suggests a closer proximity to the surface) and local configuration, as confirmed by the STM investigation in the following.

Based on the acquired XPS data we can exclude molecular decomposition because, (i) the overall intensity ratio between the N 1s and C 1s peaks (Fig. 3b, inset) remains constant up to 200 °C, which indicates that the atomic stoichiometry is preserved, and (ii) both N 1s and C 1s peaks vanish completely at 250 °C, which indicates a simultaneous desorption of the chemical species. The thermal stability up to 200 °C is comparable to NHCs measured using the same setup.^30^ However, NHC showed gradual decomposition upon annealing, which was not detected for MIC 1, thus indicating their improved molecular stability.

The thermal stability of MIC 1 is slightly higher than the results that were previously reported^42^ and can be attributed to the vacuum deposition method used in this work, which may lead to improved self-assembly pattern.^60^ However, the thermal stability is lower overall than demonstrated for NHCs with a comparable structure,^2^ possibly due to different decomposition pathways. Interestingly, the stability is comparable to that of more compact NHCs lacking an aromatic ring on the backbone,^30^ highlighting the effect of sterics and electronic stabilization on monolayer stability rather than the strength of the NHC-metal bond. The spectroscopic evidence can not lead to a definitive conclusion about the behaviour of the molecules following their annealing to 200 °C and dealkylation of the nitrogen atoms can be a plausible molecular decomposition pathway.

**Fig. 4:**
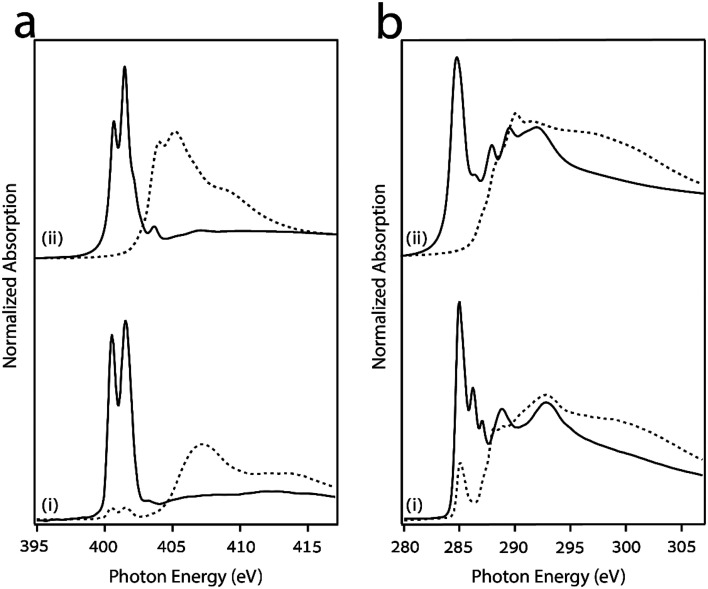
(a) Nitrogen and (b) carbon K-edge NEXAFS spectra of MIC 1, (i) measured and (ii) simulated at p- and s-polarizations (solid and dotted lines, respectively). Theoretical energies are evaluated by a constant that is adjusted by aligning to measured features.

Polarized nitrogen and carbon K-edge NEXAFS measurements were conducted (Fig. 4a(i) and b(i), respectively) to identify the adsorption geometry of the molecules, which can be deduced by comparing the intensities of the absorption spectra measured in s- and close to p-polarization, marked by dotted and solid lines, respectively. For comparison, the DFT-simulated nitrogen and carbon K-edge NEXAFS spectra for gas-phase MIC 1 in a coplanar conformation of the phenyl and triazole rings are presented in panel (ii) of Fig. 4a and b, showing a good agreement with the measured spectra. The decomposition of contributions from inequivalent N and C atoms is illustrated in Fig. S8.

The nitrogen K-edge NEXAFS spectrum in p-polarization (Fig. 4a(i)) displays two major and sharp resonances, located at 400.5 and 401.5 eV. Both peaks correspond to N 1s → π*-symmetry transitions of the triazole ring, whereas the low energy peak is associated with the central nitrogen atom N1, and the high energy peak is associated with the N2 and N3 atoms (those bound to the carbon atoms, see Fig. S8).^61^ In s-polarization, both resonances almost vanish indicating an orientation of the triazole ring closely parallel to the surface. The very small residual intensity, ∼5%, might be partly attributed to molecular defects and/or to a very small tilt off the surface. Above the ionization threshold (∼404 eV), two very broad σ*-symmetry resonances were detected in the s-polarization spectrum at ∼407 and 413–414 eV. The opposite dichroism in the σ* region further suggests a preference for a flat-lying orientation relative to the surface.

The measured carbon K-edge NEXAFS spectrum (Fig. 4b(i)) displays a more complex distribution of resonances: three well resolved peaks can be detected in p-polarization below the ionization threshold at 285.0, 286.2, 287.0 eV, as well as a fourth and a fifth peak at ∼288.8 and 292.8 eV above the ionization threshold. All these resonances correspond to C 1s → π*-symmetry transitions localized on the triazole and the aromatic ring, whereas the isopropyl groups only yield weak and broad contributions at and above the ionization threshold. According to the spectral contribution analysis in Fig. S8, both the lowest energy and highest energy π*-symmetry resonances are mostly contributed by the phenyl atoms. Consequently, the large residual intensity of the leading resonance at 285.0 eV observed in s-polarization (∼25%) can be effectively associated with a tilt off the surface by 35° of the phenyl ring, indicating a non-perfect coplanarity of the adsorbed species.

**Fig. 5:**
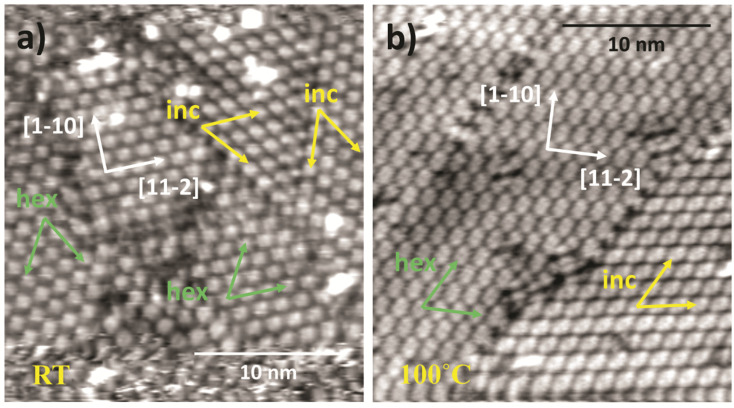
STM imaging of MIC 1 (a) after deposition at RT (130 K, −1340 mV, 210 pA) and (b) after annealing to 100 °C (130 K, −1340 mV, 220 pA); (a) and (b) images have been scanned at 30° different orientation; the orientation of the two different phases hex and inc are represented by the corresponding vectors in green and yellow, respectively.

STM imaging of as-deposited MIC 1 on Au(111) (Fig. 5) showed the formation of characteristic molecular features of square/rhombus shape that self-assemble into ordered domains. At first glance one may appreciate the coexistence of two different symmetry phases, each one displaying its three-fold rotated domains. The two phases display a similar pattern, but they show a clear, albeit small misalignment of the molecular stripes. We can distinguish a hexagonal phase (hex) formed by molecular features aligned along the substrate <11−2> directions, and a distorted and incommensurate one (inc), which deviates from the <11−2> direction by 7–8°. These two phases remain even after sample annealing to 100 °C, simply displaying a larger extension of molecular domains, as shown in Fig. 5b.

**Fig. 6:**
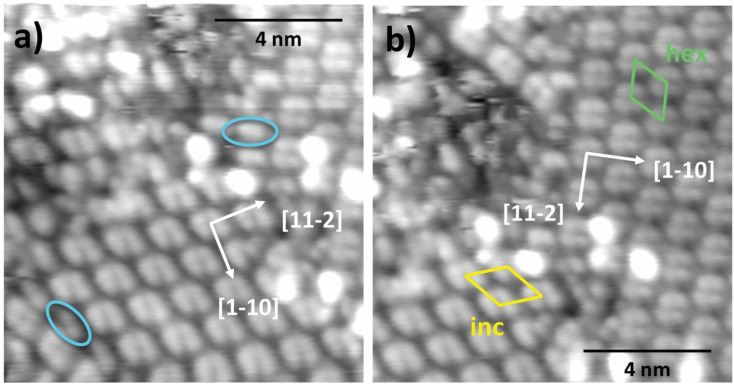
STM imaging of MIC 1 after deposition at RT (130 K, 1340 mV, 200 pA); (a) the blue ovals highlight the isolated monomers with characteristic bean shape that are found at the domain walls between different phases; (b) the unit cells of the hex (parallel to the [1−12] directions) and inc phases are represented in green and yellow, respectively.

A closer inspection of the molecular features within the two phases (Fig. 6) reveals that each molecular feature displays the same shape in both phases and appears to be formed by the pairing of two separate subunits. The latter display a characteristic bean shape and can be found also as isolated species at domain walls and in defected regions of the surface, as highlighted by markers in Fig. 6a. The lateral size of these bean-shape features is compatible with that of a flat MIC 1, thus the hex and inc phases are both formed by MIC 1 dimers displaying the same pairing geometry, as can be best appreciated in Fig. 6b. The lattice vector of the hex-phase is found in the range of 12.5 ± 0.2 Å, *i.e.* very close to an half integer multiple (5/2) of the Au(111) periodicity along the <11−2> direction and suggests a (5/2√3 × 5/2√3)-*R*30° periodicity, corresponding to a molecular density of 1.5 molecules per nm^2^. This value is significantly higher than values obtained for bulky NHCs with *tert*-butyl groups (0.57 molecules per nm^2^) which were shown to adopt a similar adsorption geometry.^62^ This value is comparable to the densities obtained for di-isopropyl-NHCs that adopted an upright adsorption configuration, which self-assembled in densities of 1.7–2.0 molecules per nm^2^.^62,63^ The high packing density is in line with the high structural order and the observed tilt of the phenyl ring.^62^ In the latter case, we remark that adjacent dimers would however display different adsorption sites (*e.g.* hollow and on-top). In this regard, we cannot exclude the presence of a tiny modulation of the surface charge corrugation, escaping the sensitivity of our STM apparatus, which would yield a higher order periodicity (at least double) with an even number of dimers in the unit cell. The determination of the lattice parameters of the inc-phase was made difficult by the small deviation of the dimer rows from the substrate symmetry directions and further hindered by the presence of the substrate herringbone reconstruction (see Experimental section for calculation details). We found a dimer spacing in the range of 13.5–14.0 Å, with an angle of 52° ± 2° between the superlattice vector, corresponding to a molecular density in the range of 1.3–1.4 molecules per nm^2^, slightly lower than in the hex-phase.

The adsorption geometry adopted by MIC 1 on Au(111) at room temperature is comparable to NHCs with di-isopropyl side groups, which are positioned flat on the Au(111) surfaces at moderate coverages, although an upright orientation was observed in higher coverages.^64^ MIC molecules are also bound to the surface *via* a gold adatom, similarly to NHCs with various structures.^1–3,63,64^ The formation of dimers, however, was not previously observed for MIC molecules.^3,64^ A similar dimer configuration was observed for NHCs functionalized with several other bulky side or backbone groups, for example NHCs with butyl groups adopted a flat lying configuration and were adsorbed as NHC–Au adatom–NHC complexes,^65–67^ implying that steric properties may play a dominant role in dimer formation also in the case of MIC 1.

**Fig. 7:**
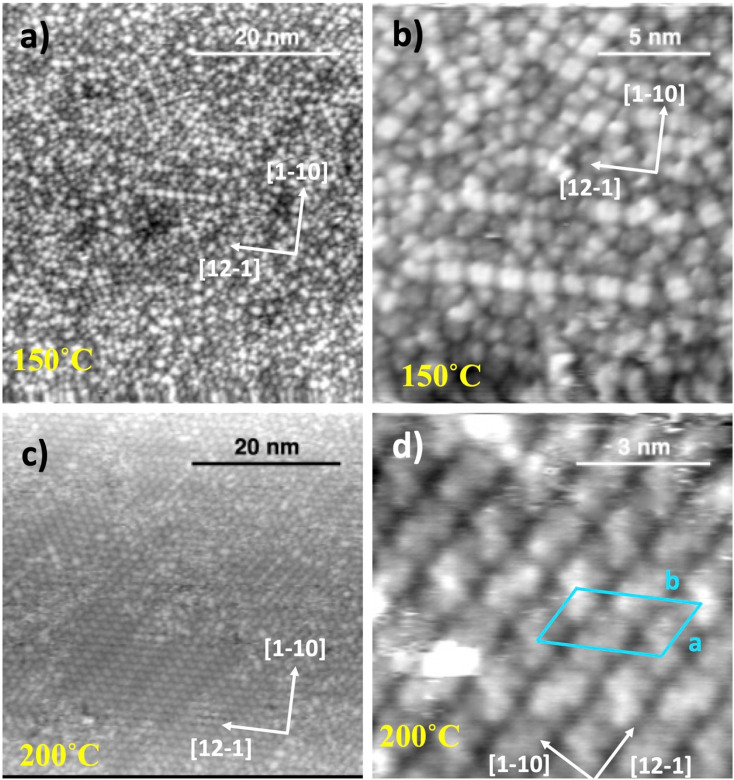
Upper panels (a) and (b): STM imaging of MIC 1 following annealing to 150 °C (130 K, +1430 mV, 300 pA); lower panels (c) and (d): same surface after annealing to 200 °C; (c) 130 K, +1400 mV, 300 pA; (d) 130 K, +1200 mV, 250 pA. The unit cell of the commensurate phase formed at 200 °C is superimposed to the molecular features in panel (d).

The SAM morphology undergoes a dramatic change upon annealing to 150 °C, where not only the long-range order is lost, but even the local structure of the molecular assembly changes. In the two upper panels of Fig. 7, one may appreciate the coexistence of residual dimeric units and new bean-shaped smaller units; both species are mostly irregularly spaced but show the tendency to align along the substrate <11−2> main symmetry directions.

The SAM evolution is completed by further annealing to 200 °C, where a new long range ordered phase is observed. The molecules now give rise to characteristic elongated features that are still dimensionally compatible with the formation of dimers displaying a chiral intramolecular contrast. Dimers with the same molecular orientation line up along one of the <11−2> directions with an in-line spacing of ∼15 Å, where adjacent rows display opposite azimuthal orientation. The observed spacing of ∼30 ± 0.5 Å between equivalent rows (15 Å between adjacent rows) is compatible with the formation of a commensurate superlattice with a (3√3 × 6√3)-*R*30° unit cell containing two dimers with opposite azimuthal orientation. This commensurate phase displays a molecular density of 1 molecule per nm^2^, which is in good agreement with the observed decrease of the C 1s and N 1s XPS intensity with respect to the RT monolayer (see Fig. 3). Phase transitions upon annealing were observed for NHCs as well. In the case of NHC with di-isopropyl side groups (iPr-NHC), mild annealing to 50 °C led to a higher structural order, and annealing to 140 °C induced a phase transition in iPr-NHC molecules, in which a dimer phase was observed following the annealing.^68^

**Fig. 8:**
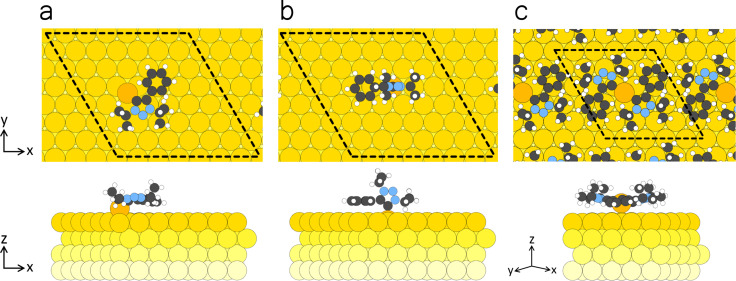
Top and side views of the following structures: (a) MIC 1 adsorbed with the binding C atom attached to the Au(111) substrate *via* an Au adatom, and (b) MIC 1 adsorbed with a carbene C atom directly attached to a substrate Au atom. (c) MIC 1–Au–MIC 1 complex on Au(111). Color code: C, gray; N, blue; H, white; Au, yellow, turning into lighter yellow while entering the substrate. A 7 × 7 4-layered supercell of Au(111) is considered with a lattice constant of 20.355 Å. Axes indicate *x* = [1−10], *y* = [11−2], and *z* = [111].

DFT structural optimizations were conducted to elucidate the adsorption geometry of the molecules and their interaction with the Au(111) surface. We first consider the adsorption of individual MICs. Following relaxation, it was observed that the optimized surface anchoring is achieved *via* the carbene carbon and not *via* the nitrogen atoms. Two possible stable configurations for the adsorption of one MIC 1 on Au(111) were considered: (1) a flat-lying configuration (Fig. 8a) in which the carbon atom is bound *via* an Au adatom, and (2) a standing configuration (Fig. 8b) in which MIC 1 is adsorbed with a vertically mesoionic ring having the carbene C atom directly attached to a substrate Au atom, while the phenyl ring is still parallel to the surface.

Adsorption energy calculations (Table S1) revealed that the molecules are preferably adsorbed *via* an adatom, with an adsorption energy difference of 0.699 eV between the two adsorption geometries. This difference, however, does not compensate for the high cost of adatom formation (0.761 eV). Thus, the formation energy of the configuration directly attached to the surface (Fig. 8b) would be favorable (−2.923 eV). However, this energy ordering is reversed for the formation of molecular superstructures as will be discussed below. It is hypothesized that the source of adatoms was in step edges, as concluded in our previous work for N-heterocyclic olefins, in which a significant indentation of monoatomic steps was observed.^30^ The results from the calculated configuration shown in Fig. 8a are in good agreement with the experimental NEXAFS measurements (Fig. 4(i)). In particular, the triazole ring was found to be parallel to the surface, whereas the phenyl ring, although leaning towards the surface, was experimentally found to be slightly tilted off the surface.

In MIC 1–Au–MIC 1 complexes on Au(111), the two molecules share the same Au atom (Fig. 8c) and thus the cost of adatom formation per molecule is reduced. Overall, the formation energy per molecule (Table S1) amounts to −3.099 eV, that is 0.176 eV more stable than for individual MIC 1. In this configuration, the Au adatom is lifted from its normal position and is positioned in between the molecules, attaining a height of 2.772 Å that is 0.771 Å higher than that of Au adatom on Au(111). This height compares well with the average height of 3.1 Å reported for the SAM of 1,3-dimethylimidazole-2-ylidene^58^ and is fully consistent with the CLS measured in the Au 4f XPS data (Fig. 2).^3,58^

The MIC 1–Au–MIC 1 complex constitutes the building block of the experimentally observed phases on Au(111) that vary in their lateral arrangement. The energy of the complex only mildly depends on the specific position and orientation over the substrate: for example, the central Au adatom can be translated to the top site at a cost of 0.01 eV per MIC, and can be rotated by 30° around the surface normal at a cost of 0.02 eV per MIC. Additional molecular dynamics simulations performed (135 K) for individual dimers at the experimental STM temperature have shown rotation and diffusion events, compatible with the flat potential energy surface (but no breakup within 20 ps). Interestingly, the triazole and the aromatic ring are observed to fluctuate around the planar position within a range of 16.7° and with an average angle of ∼13° and ∼8°, respectively (Fig. S9); however, this effect is too small to justify the NEXAFS dichroism of Fig. 4b, spectrum (i), that requires a tilt angle of about 30°. We therefore speculate that the observed tilting of the aromatic ring is an effect of the interaction between facing MIC 1–Au–MIC 1 complexes when condensed structures are formed. For example, structural optimization of a free-standing layer of MIC 1–Au–MIC 1 complexes with a 5/2√3 × 5/2√3 unit cell as in the hex phase results in a tilt angle reaching 40°, in good agreement with NEXAFS results. We remark that theoretical analysis of the substrate of the two phases is hampered by the fact that an exceedingly large-scale model is required to account for the incommensurate (inc) and the commensurate (hex) phases (a model for the (5√3 × 5√3)-*R*30° involves more than 500 atoms).

**Fig. 9:**
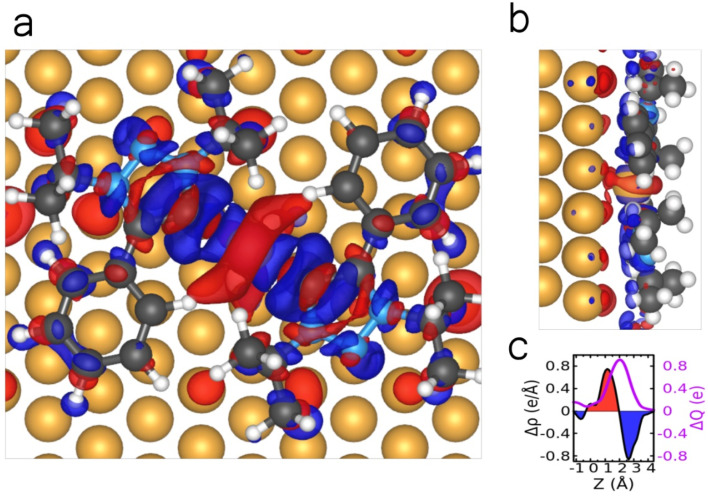
(a) Top and (b) side views of the electron density difference for the adsorption of MIC 1–Au–MIC 1 complex on Au(111). This is calculated as Δ*ρ* = *ρ*_mol1+mol2+Au/Au(111)_ − *ρ*_mol1_ − *ρ*_mol2_ − *ρ*_Au_ − *ρ*_Au(111)_*i.e.*, the difference between the electron density of the whole system and that the two MIC 1 molecules, of the adatom, and of the substrate. The red and blue isosurfaces (isovalue = 0.0088 Å^−3^) represent electron density increase and decrease, respectively. (c) Planar-averaged electron density difference Δ*ρ*(*z*) (black) and its integral in *z*, that is the amount of transferred charge across a plane at distance *z*, Δ*Q*(*z*) (magenta). Atomic color code as in Fig. 8.

For the same reasons, we simulate a model 5 × 5 superstructure to determine the electronic properties of the adsorbed MIC 1–Au–MIC 1 complex: this constitutes a viable way to attain within a commensurate unit cell a surface density reasonably close to the observed ones, namely 1.1 molecules per nm^2^. We first analyze the interface charge transfer and show in Fig. 9 the electron density difference (Δ*ρ*) upon the adsorption of the MIC 1–Au–MIC 1 complex on the Au(111) substrate with respect to the sum of isolated substrate, MIC 1 molecules, and adatom. This shows that electrons have accumulated at the interface between the surface and the molecules, at the expense of about 1 electron coming from the central regions around the Au adatom and carbene C atom: the latter were especially electron rich and donate density to the Au adatom in forming a hypothetical dimer in the gas phase. That density is eventually displaced towards the surface in adsorbing the dimer. This is detailed in Fig. S10, where this electron density displacement is analyzed in terms of more elemental contributions. Overall, the planar average density displacement and its integration (Fig. 9c) indicate that about 0.8 electrons are displaced from the molecules to the interface region.

**Fig. 10:**
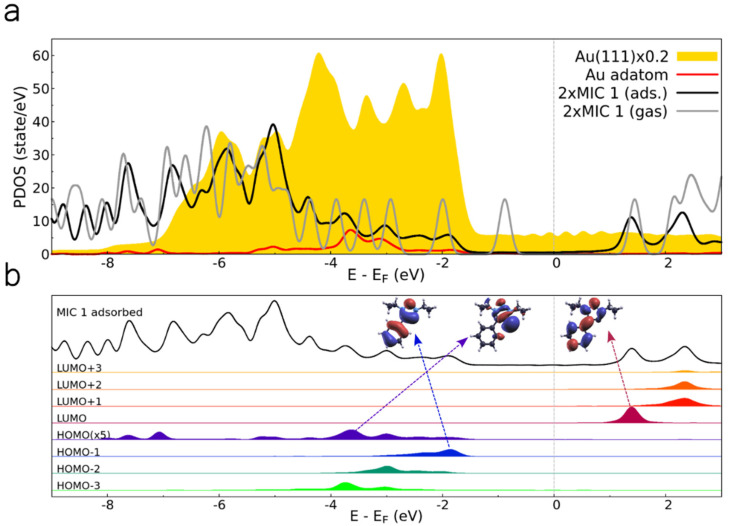
Density of states (MOPDOS) analysis of MIC 1–Au–MIC 1 complex. (a) DOS projected onto the Au(111) surface (filled yellow curve), Au adatom (red curve), and NHC MIC 1 molecules (gray and black curves for adsorbed and gas phase, respectively). The Fermi level (*E*_F_) is set to 0 eV. (b) Molecular-orbital projected DOS of an adsorbed NHC MIC 1 molecule within the dimer, showing contributions from frontier molecular orbitals. For clarity, the HOMO DOS is multiplied by a factor 5. Insets display wavefunction amplitudes of selected molecular orbitals of MIC 1 in the gas phase (HOMO−1, HOMO, and LUMO), indicating their energy alignment and hybridization with the substrate.

The projected density of states (PDOS) for the MIC 1–Au–MIC 1 complex is presented in Fig. 10. Upon adsorption (black line), molecular orbitals broaden and shift with respect to the free molecule case (gray) due to hybridization with the metal surface states. Interestingly, the highest occupied molecular orbital (HOMO) of the free MIC 1 disappears in the adsorbed DOS. For further details, Fig. 10b analyzes the DOS by its projection onto atomic orbitals,^69^ focusing on the frontier orbitals. Notably, specific molecular states exhibit significant overlap with the Au(111) energy levels, resulting in a strong energy broadening. This phenomenon strongly depends on the specific orbital. As can be seen in Fig. 10b, the HOMO and HOMO−1 exhibit strong electron density at the carbene C atom, enhancing overlap with metal states especially for the σ orbital HOMO, interacting most strongly with the Au adatom, which is spread across several eVs.

## Conclusions

This study provides an in-depth investigation into the self-assembly pattern of 1,2,3-triazole-based mesoionic carbenes (MICs) on Au(111), uncovering their adsorption geometry, thermal stability, and self-assembly patterns. By combining synchrotron-based techniques such as XPS, NEXAFS, and STM imaging with DFT calculations, we elucidated how the electronic properties of MICs differentiate them from traditional NHCs. It is identified that MICs are self-assembled in a flat-lying adsorption configuration, stabilized by molecule-adatom interactions. The self-assembled MIC monolayers formed highly ordered, rhombus-like patterns on Au(111), with molecule-adatom-molecule motif, enabling long-range structural order and a high density of 1.4–1.5 molecules per nm^2^. The interaction between MICs and adatoms induced significant changes to the electronic structure of the complex. The mesoionic nature of MICs contributed to their thermal stability, withstanding temperatures up to 200 °C without significant molecular decomposition. Unlike many NHC-based systems, which often undergo gradual thermal degradation, triazole-based MICs desorb without decomposition, highlighting their chemical stability. In particular, the dimer motif gives rise to full wetting long range ordered compact phases up to the limit of desorption temperature. These findings support the potential incorporation of triazole-based MIC monolayers in various devices that operate under or require high-temperature processing.

## Conflicts of interest

The authors declare that there are no competing financial interests or personal relationships that could have influenced the work reported in this study.

## Supplementary Material

NR-017-D5NR02802G-s001

## Data Availability

Data supporting the findings of this study are provided in the SI: experimental and synthetic details, additional computational and XPS results, and details about STM calibration. See DOI: https://doi.org/10.1039/d5nr02802g. All raw spectroscopic data are available from the authors upon a reasonable request.
