# Supplementary material for: Self-assembled monolayers of mesoionic triazolylidene dimers on Au(111)
Source: Nanoscale. 2025 Oct 24;17(43):25213–26. doi: 10.1039/d5nr02802g (PMC12550663; doi:10.1039/d5nr02802g)
Supplement: NR-017-D5NR02802G-s001 [file NR-017-D5NR02802G-s001.pdf]

Supplementary Information (SI) for Nanoscale.
This journal is © The Royal Society of Chemistry 2025

Supporting Information

Mesoionic Triazolylidene Dimers Form Dense and Thermally Robust Monolayers on Au(111)

Iris Berg,[a],+ Luca Schio,[b],+ Masoumeh Alihosseini,[c] Justus Reitz,[d] Elena Molteni,[c] Shuangying Ma,[c] Carolina Gutiérrez Bolaños, [e] Andrea Goldoni,[e] Cesare Grazioli,[b] Max M. Hansmann,[d] Guido Fratesi,[c],* Luca Floreano,[b],* and Elad Gross, [a],*

+ These authors contributed equally to this work

[a] I. Berg, Prof. E. Gross

Institute of Chemistry and The Center for Nanoscience and Nanotechnology, The Hebrew University, Jerusalem 91904, Israel

[b] Dr. L. Schio, Dr. C. Grazioli, Dr. L. Floreano

CNR-IOM, Istituto Officina dei Materiali, Basovizza SS-14, Km 163.5, Trieste 34149, Italy

[c] Dr. M. Alihosseini, Dr. E. Molteni, Dr. S. Ma, Prof. G. Fratesi

Dipartimento di Fisica ``Aldo Pontremoli'' Università degli Studi di Milano, Via Celoria 16, 20133 Milano, Italy

[d] J. Reitz, Prof. M. M. Hansmann

Technische Universität Dortmund, Fakultät für Chemie und Chemische Biologie, Otto-Hahn-Str. 6, 44227 Dortmund, Germany

[e] C. Gutiérrez Bolaños, Dr. A. Goldoni

Elettra-Sincrotrone Trieste S.C.p.A, Basovizza SS-14, Km 163.5, Trieste 34149, Italy

Corresponding author

* Guido Fratesi: guido.fratesi@unimi.it; Luca Floreano: floreano@iom.cnr.it; Elad Gross: elad.gross@mail.huji.ac.il

Experimental information

The synthesis of the 1,2,3-triazolium triflate salt (**1-OTf**) was reported previously.[1] The bicarbonate precursor was prepared according to an adapted literature procedure.[2]

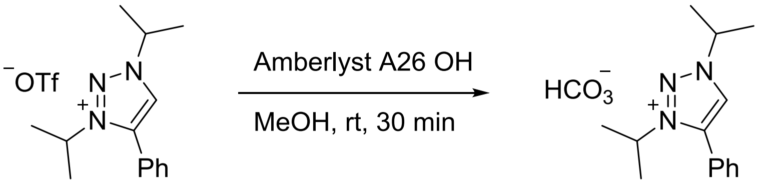

Amberlyst A-26(OH) (2.00 g, 4.2 meq/g, 2.0 eq.) was suspended in H_2O (10 mL). Under thorough stirring, CO_2 was bubbled through the suspension for 1.5 h. The Amberlyst was then filtered off and washed with MeOH (2 x 5 mL). The triazolium triflate salt (800 mg, 2.11 mmol, 1.0 eq.) dissolved in MeOH (10 mL) is added to the Amberlyst and the suspension was stirred under bubbling with CO_2 for 1 h. The beads were filtered off, rinsed with MeOH (2 x 5 mL) and the filtrate was concentrated. The residue was triturated with acetone (2 mL) and then precipitated by adding Et_2O (50 mL). The precipitate was filtered, dried and washed with acetone (2 x 1 mL) and dried under reduced pressure to afford the desired triazolium bicarbonate) (**1-HCO$_3$**) (0.42 mg, 1.42 mmol, 68 %) as a colorless solid.

The carbene ^{13}C NMR signal and the corresponding ^{1}H NMR signal could not be observed due to the fast exchange with the deuterated solvent.

^{1}H NMR (501 MHz, $CDCl_3$, 298 K) δ [ppm] = 7.66 – 7.58 (m, 2 H, Ph-CH), 7.58 – 7.49 (m, 3 H, Ph-CH), 5.47 (hept, J = 6.7 Hz, 1 H, CH(CH$_3$)$_2$), 4.95 (hept, J = 6.6 Hz, 1 H, CH(CH$_3$)$_2$), 1.74 – 1.65 (m, 6 H, CH(CH_3)$_2$), 1.62 (d, J = 6.6 Hz, 3 H, CH(CH_3)$_2$); **^{13}C NMR** (126 MHz, $CDCl_3$, 298 K) δ [ppm] = 160.5 (HCO_3^-), 141.9 (triaz-C$_q$), 131.7 (Ph-CH), 129.9 (Ph-CH), 129.8 (Ph-CH), 122.6 (Ph-C$_q$), 58.8 (CH(CH$_3$)$_2$), 55.1 (CH(CH$_3$)$_2$), 22.8 (CH(CH$_3$)$_2$), 22.4 (CH(CH$_3$)$_2$); **ATR-IR** [cm^{-1}]: 2962, 1649, 1468, 1451, 1390, 1344, 1309, 1278, 1223, 1059, 1036, 849, 803, 778, 770, 755, 722, 698, 441; **HR-MS-ESI(+)** m/z calcd. for $C_{14}H_{20}N_3^+$ [M-HCO_3^-]$^+$ 230.1652; found 230.1658. NMR, ATR-IR and MS graphs are presented in Figure S1-S5.

Thermogravimetric analysis measurements (Figure S6) were performed on a Discovery SDT 650 instrument from TA Instruments under a constant nitrogen flow of 100 mL/min. For the

measurement a small amount (~ 6 mg) of the powdered sample was placed in a 90 μL alumina crucible and heated from 40 °C to 200 °C with a constant heating rate of 10 K/min.

Au (111) single crystal with a diameter of 10 mm (purchased from SPL) was cleaned under ultrahigh vacuum conditions by three consecutive cycles of sputtering (2×10^{-6} torr Ar; 1.5 keV; 20 min) and subsequent annealing to 500 °C (two thermocouples in direct contact with the sample). The effectiveness of the cleaning procedure was validated by monitoring the C1s XPS signal (Figure S11).

Vapor-deposition of MIC **1** was performed in a UHV chamber at ALOISA beamline. The molecules were housed in a home-made Knudsen cell (boron nitride crucible with thermocouple inside). The evaporation temperature was raised to 358 K and the molecules were dosed into the UHV chamber, inducing an increase in the back-pressure in of ca. 10^{-8} mbar. The Au surface was kept at room temperature (RT) and exposed to the evaporated molecules for 20 minutes. Several consecutive deposition cycles were conducted and N1s and C1s XPS measurements were performed after each cycle. These measurements did not show noticeable changes in the signal amplitude, thus validating the exclusive formation of a saturated monolayer on the surface at RT.
Synchrotron radiation XPS measurements were measured with the sample at grazing incidence (α = 4.0°) in Transverse Magnetic, TM, polarization (close to p-polarization). The spectra, taken with photon energy of 515 eV and 160 eV (overall energy resolution of 160 and 110 meV, respectively), were measured in close to normal emission (90°-α) by a hemispherical electron analyzer with an angular acceptance angle of 2°. The binding energy (BE) was calibrated by the fermi energy. All spectra were corrected by subtracting a Shirley-type background. Analysis of the XPS peaks and their fitting was performed using CasaXPS software.

X-ray absorption spectra measurements were taken in partial electron yield using a channeltron detector equipped with a front grid biased at negative voltage (250 V and -370 V for the C and N *K*-edge spectra, respectively) to filter out the low energy secondary electrons. The NEXAFS spectra at the nitrogen and carbon K-edge were measured with the resolution set to ~80 meV while keeping the sample at a constant grazing angle of 6°. The orientation of the surface with respect to the photon beam polarization was changed from Transverse Electric, TE, polarization (s-polarization) to TM polarization (close to p-polarization) by rotating the sample coaxially to the photon beam axis. NEXAFS spectra were reported in the form of a normalized

absorption amplitude ($I_{(E)} = I/I_{(reference)}$), using NEXAFS measured on Au (111) as a reference ($I_{(reference)}$). Tilt angle (θ), relative to the surface plane, was calculated based on the following formula (for three-fold and higher symmetry substrate) : $\tan(\theta) = \sqrt{\frac{2I_s}{I_p}}$ in which I_s and I_p represent the s-polarized and p-polarized signal amplitudes, respectively.[3]

Scanning tunneling microscopy (STM) measurements were performed at the CNR-IOM/Elettra joint laboratory for microscopy (OSMOS) in Trieste with an Aahrus type microscope (model 150 by SPECS) under Ultra-High Vacuum (UHV). The topographic images were collected by operating the STM at a typical sample temperature of 130-150 K (liquid nitrogen cooling). The bias voltage is referenced to the sample. See Figure S12 for STM calibration details.

The surface density of the molecules was derived by assuming a nominal Au periodicity of 2.88 Å. For the hexagonal phase, the unit cell was modeled as a rhombus with 12.5 Å sides and an internal angle of 60°, yielding an area of 1.35 nm^2 and accommodating two molecules, corresponding to a density of approximately 1.5 molecules/nm^2. In the incommensurate phase, the unit cell was approximated as a lozenge with an internal angle of 52° and side lengths ranging from 13.5 to 14.0 Å. These geometries correspond to surface areas of 1.44 and 1.54 nm^2, giving estimated densities of ~1.4 and ~1.3 molecules/nm^2, respectively. For the annealed phase, a parallelogram-shaped unit cell with sides of ~15 Å and ~30 Å and an internal angle of 60° yields an area of 3.88 nm^2. This unit accommodates four molecules (arranged as two dimers), resulting in an estimated surface density of ~1.0 molecule/nm^2.

Ab-initio calculations within the framework of Density Functional Theory (DFT) were performed using the Quantum ESPRESSO package.[4,5] The exchange-correlation functional was treated using the generalized gradient approximation (GGA) as proposed by Perdew, Burke, and Ernzerhof (PBE),[6] along with ultrasoft pseudopotentials.[7] To account for van der Waals interactions, the geometries obtained using GGA-PBE were further optimized by incorporating Grimme dispersion corrections (PBE-D3).[8]

The substrate was modeled using a slab approach consisting of four Au(111) layers. 7×7 and 5×5 supercells were employed for single-molecule adsorption and dimer systems, with 4×4 and 6×6 Gamma-centered Monkhorst-Pack grid used for Brillouin zone sampling, respectively. Kinetic energy cutoffs were set to 40 Ry for the plane-wave expansion of the wave function and 200 Ry for that of the charge density, as designed for GBRV pseudopotentials.[7]

During structural optimization, the bottom two layers were fixed, while the remaining layers were allowed to fully relax together with the adsorbates. The XPS is determined based on the core level shifts (CLS) between distinct nitrogen and carbon atoms through self-consistent calculations using pseudopotentials, which are generated with a 1s full core hole (FCH) at the specified atomic site.[9,10] NEXAFS spectra, within the half-core-hole (HCH) approach,[11,12] are simulated using the xspectra code in Quantum-ESPRESSO.[13] Contributions from different atoms of the same species are summed as described previously.[14,15] Since the pseudopotential method does not provide absolute transition energies, the simulated spectra are aligned with experimental data.[16]

Molecular dynamics simulations are executed with the semi-empirical GFN1-xTB[17] method implemented in CP2K[18] software, including Van der Waals and dipole corrections. We performed equilibrium simulations with an NVT ensemble at 135 K, with a time step of 1 fs, for a duration of 20 ps. During the MD simulations, the first layer of the Au(111) surface (out of two) was allowed to move together with MICs and adatoms.

Table S1: Structural and energetic parameters of optimized configurations of MIC **1** / adatom/Au(111) and MIC **1**/Au(111) substrate (4 layers, 7x7 supercell, 4x4x1 k-point mesh). The adsorption bond length is the shortest carbene C-Au distance; the heights are referred to the Au(111) topmost plane.

**Configuration**	**Adsorption bond length (Å)**	**Carbene C height (Å)**	**Adatom height (Å)**	**Adsorption energy/molecule (eV)**	**Formation energy/molecule* (eV)**
MIC **1** / adatom / Au(111): to bind with C	2.050	3.139	1.993	-3.622	-2.861
MIC **1** vertical on C, no adatom	2.112	2.411	-	-2.923	-2.923
MIC **1** - adatom MIC **1** / Au(111): (dimer)	2.061	3.072	2.747	-3.479	-3.099
MIC **1** - adatom (fixed on top site) MIC **1** / Au(111): (dimer)	2.061	3.175	2.954	-3.470	-3.089
MIC **1** - adatom MIC **1** / Au(111): (dimer 30° rotation)	2.065	3.112	2.791	-3.460	-3.080

*The formation energy includes the energy cost of adatom formation.

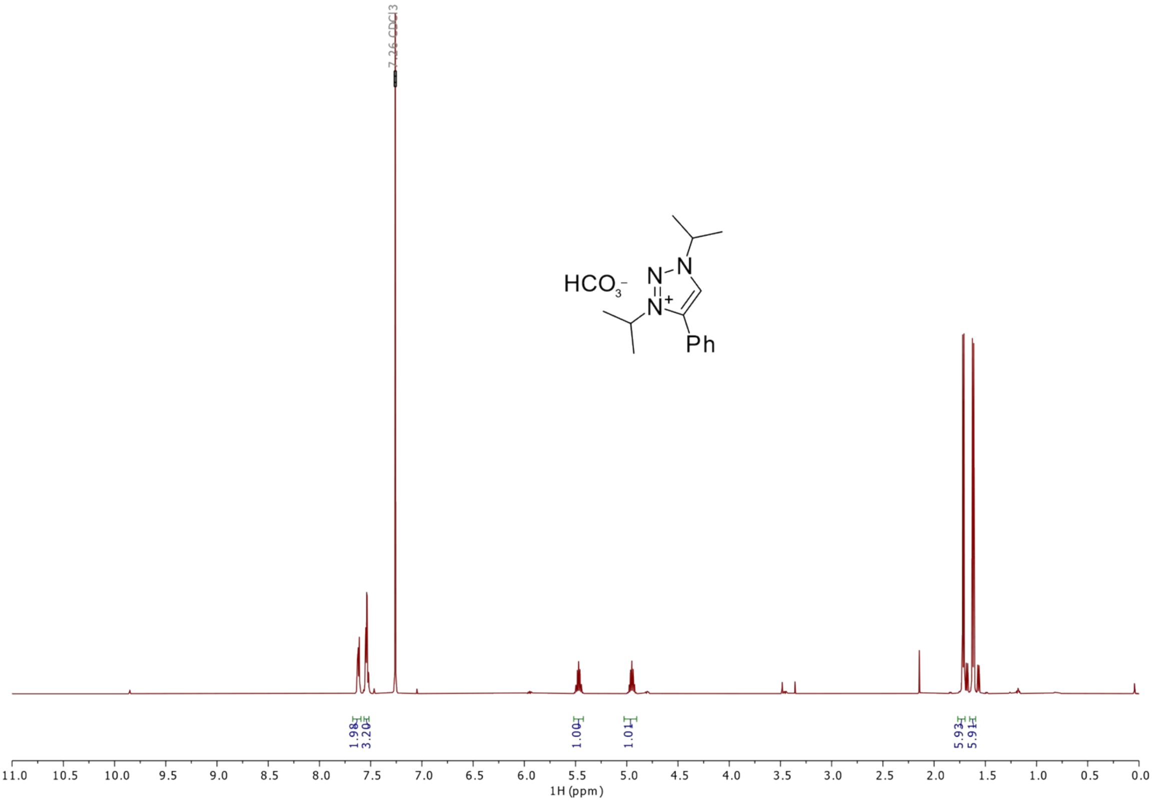

Figure S1: [1]**H** NMR spectrum (501 MHz, $CDCl_3$, 298 K) of triazolium bicarbonate (**1-HCO$_3$**).

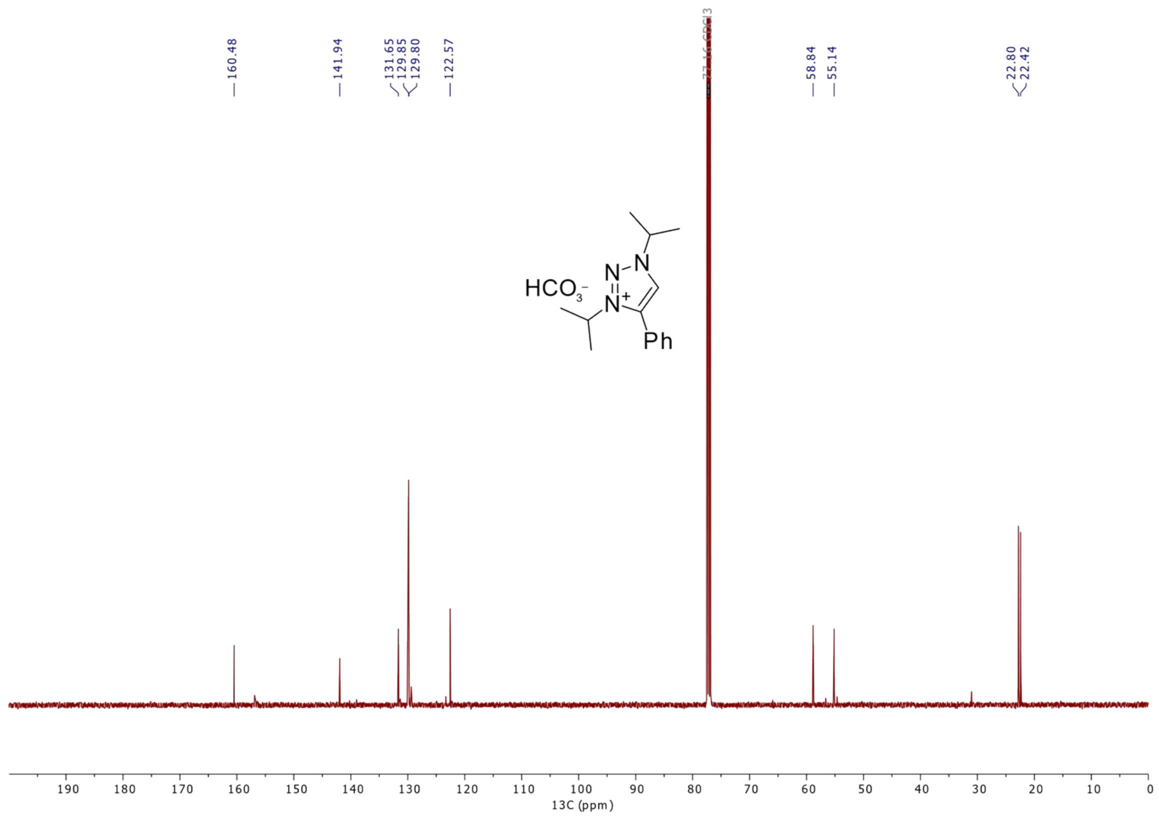

Figure S2: ^{13}**C** NMR spectrum (126 MHz, $CDCl_3$, 298 K) of triazolium bicarbonate (**1-HCO$_3$**).

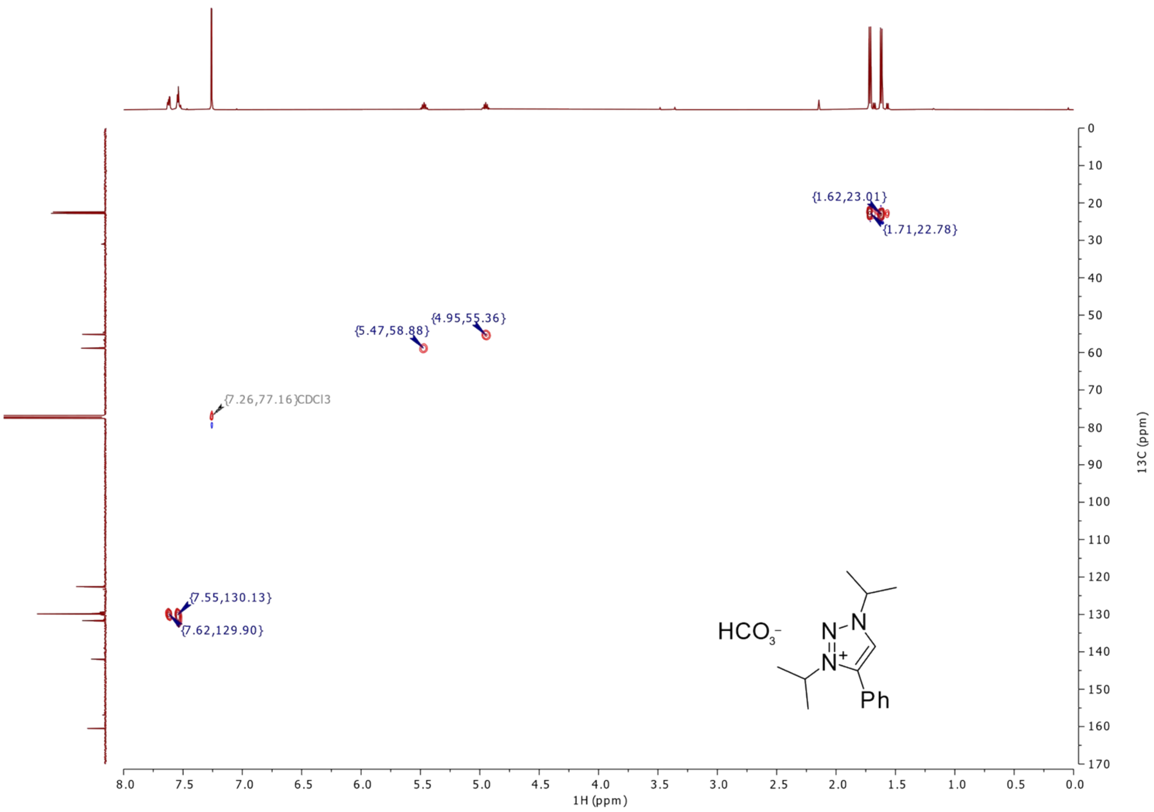

Figure S3: ^{1}H/^{13}C HSQC NMR spectrum (501/126 MHz, $CDCl_3$, 298 K) of triazolium bicarbonate (**1-HCO_3**).

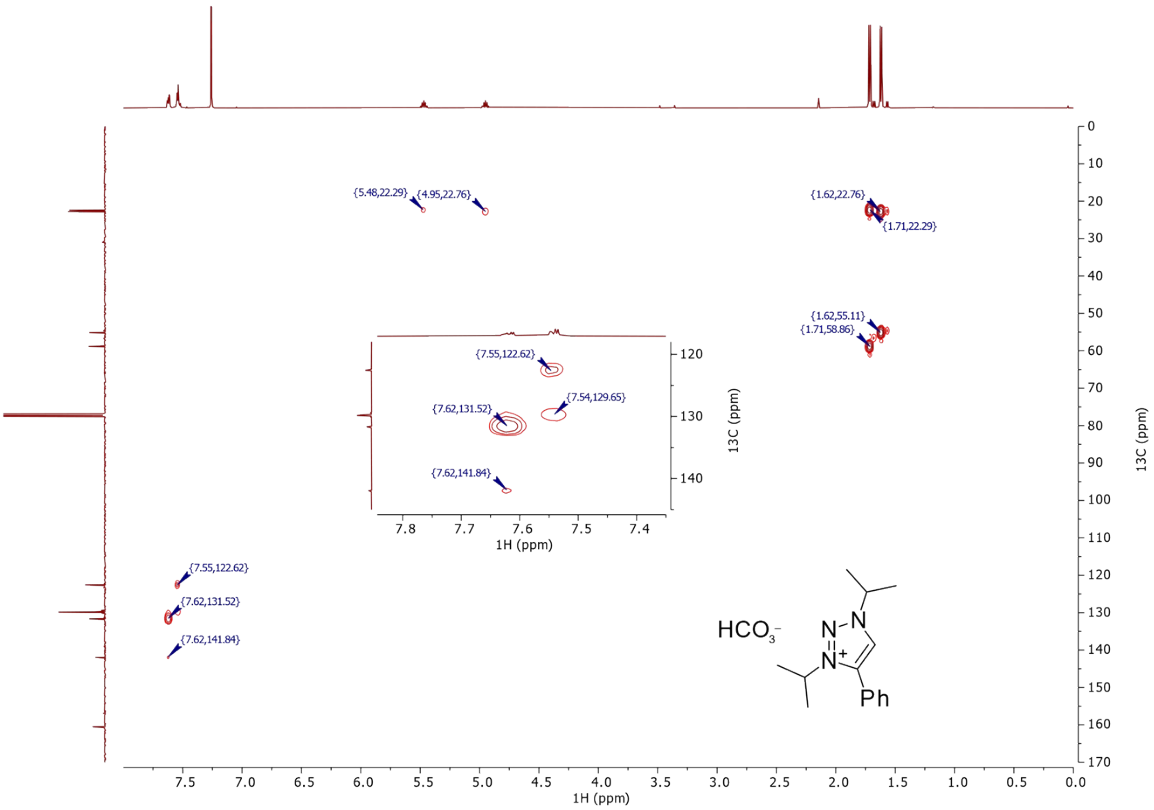

Figure S4: ^{1}H/^{13}C HMBC NMR spectrum (501/126 MHz, $CDCl_3$, 298 K) of triazolium bicarbonate (**1-HCO_3**).

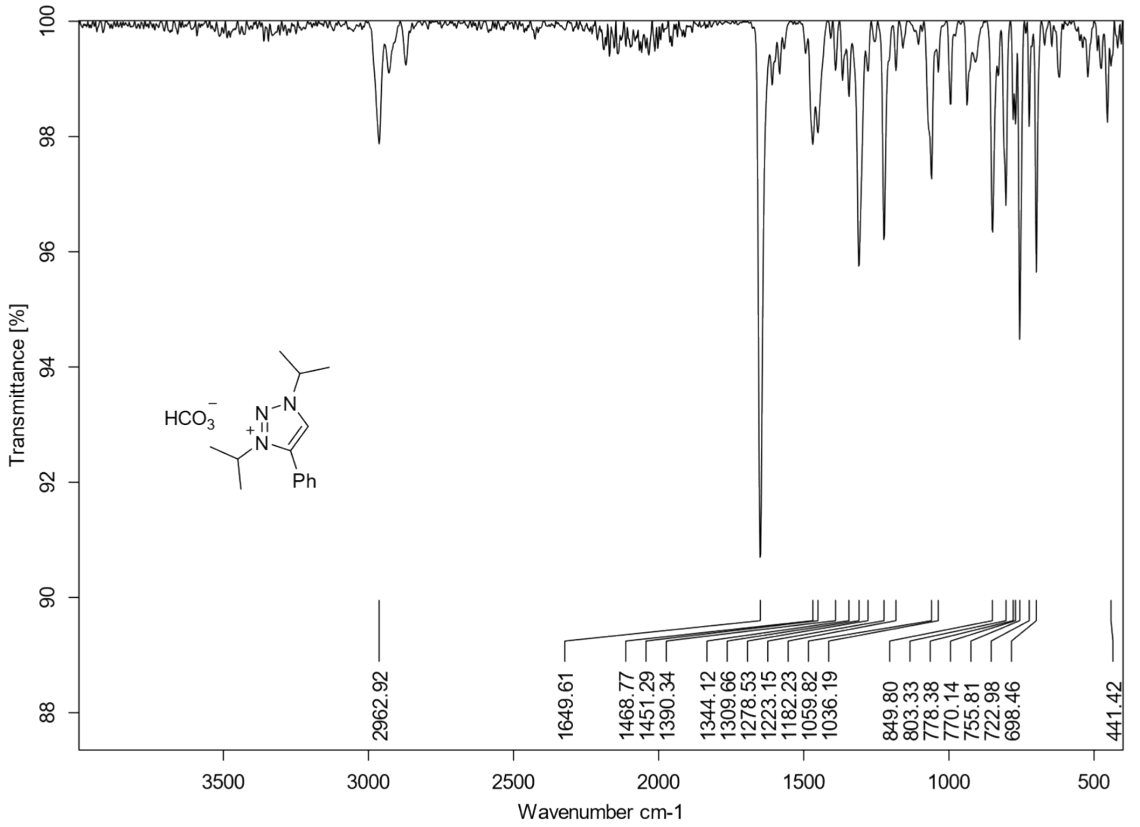

Figure S5: ATR-IR spectrum of triazolium bicarbonate (**1-HCO_3**).

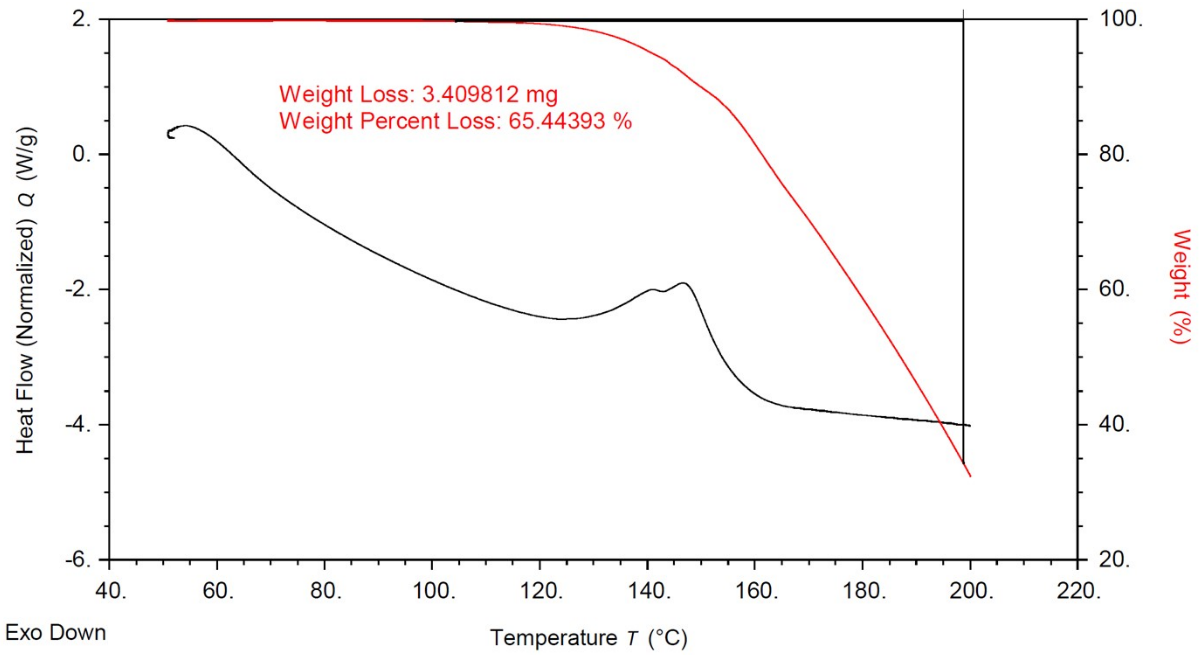

Figure S6: TGA of triazolium bicarbonate (**1-HCO_3**).

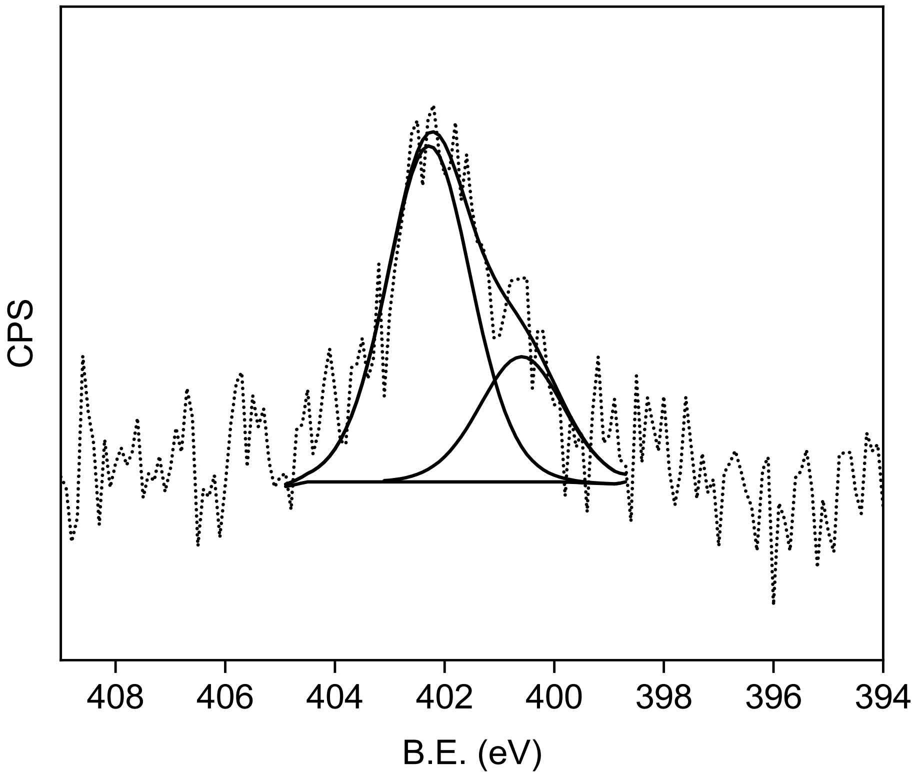

Figure S7: N1s XP spectra of MIC **1** that was deposited on Au film by base-induced deprotonation.

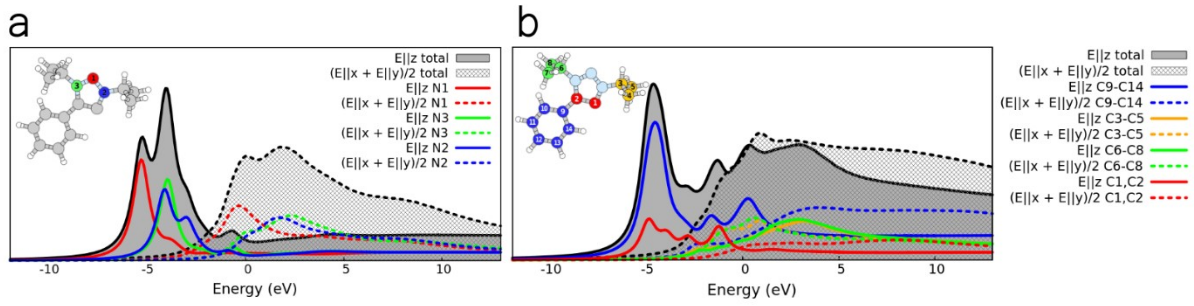

Figure S8: Simulated nitrogen K-edge (a) and carbon K-edge (b) NEXAFS spectra for gas-phase MIC **1**. The distribution of atoms is shown with colors matching those of the atoms in the inset.

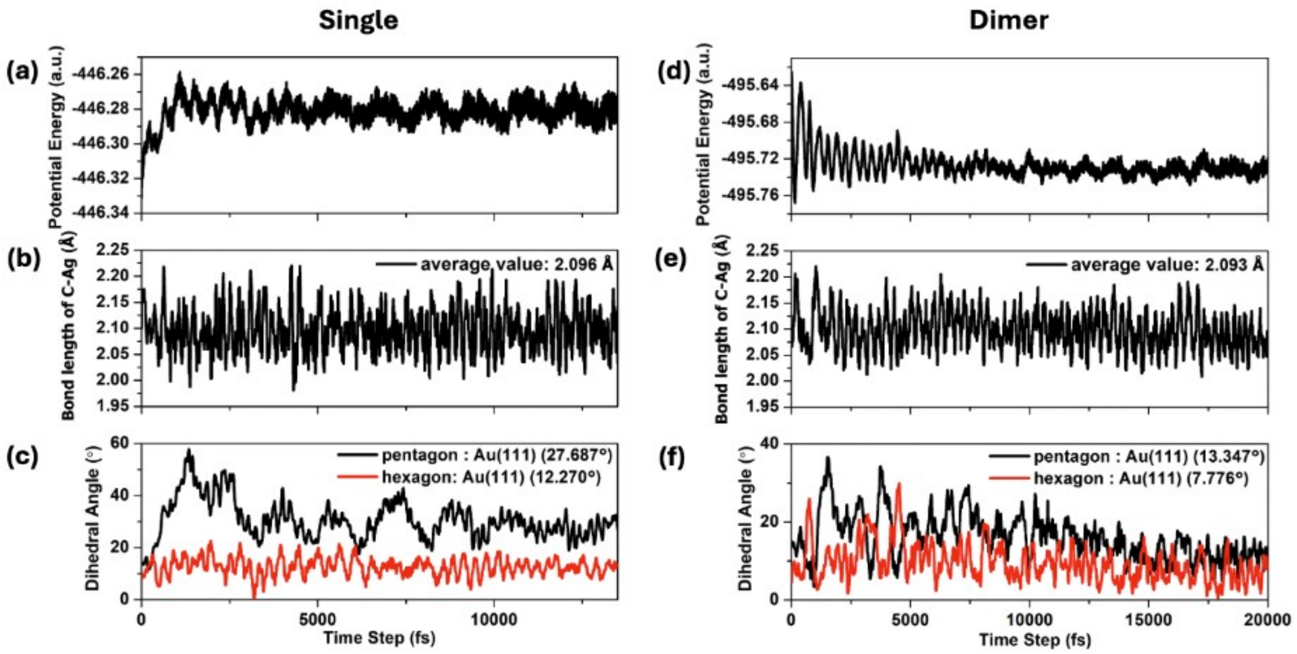

Figure S9: The potential energy (a, d), distance between nearest C and Au adatoms (b, e), and dihedral angles between the plane formed by the triazole ring (pentagon) or by the aromatic one (hexagon) (c, f) of single and dimer MIC **1** molecules (see Figure 8a and Figure 8c in the main text) on Au(111) as a function of time during a molecular dynamics simulation. Average values are evaluated after an equilibration step of 5 ps.

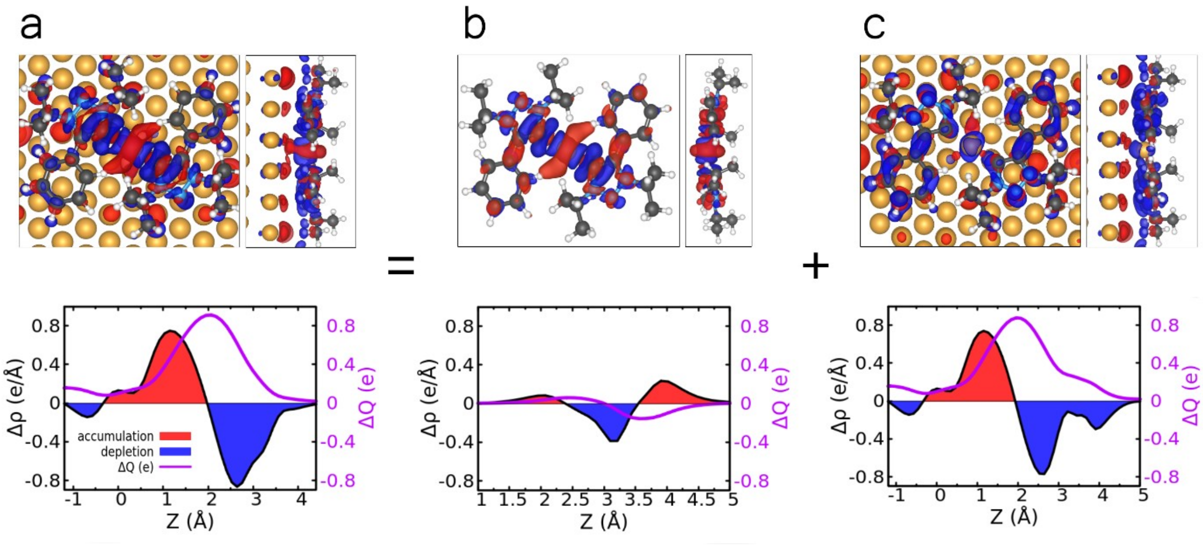

Figure S10: 3D top/side views with isosurface (top) and plane-averaged results (bottom) of the charge density difference for the MIC **1**-Au-MIC **1** complex (as in Figure 9 of the main text). (a) Total charge redistribution upon adsorption, decomposed into (b) the contribution from the isolated molecules binding to a Au atom and (c) the adsorption of the resulting MIC 1-Au-MIC 1 complex on Au(111). The regions of electron density accumulation and depletion are depicted in red and blue, respectively (isovalue = 0.0088 $Å^{-3}$).

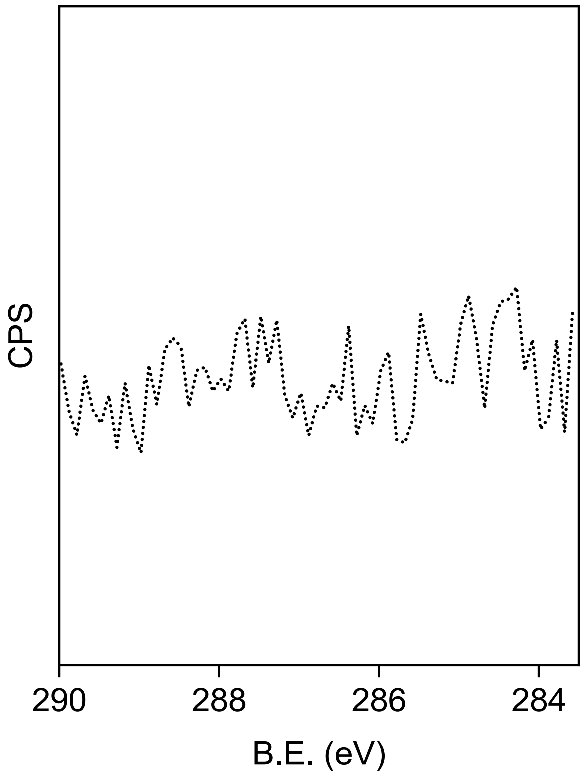

Figure S11: C1s XPS spectrum of Au (111) following sputter-annealing.

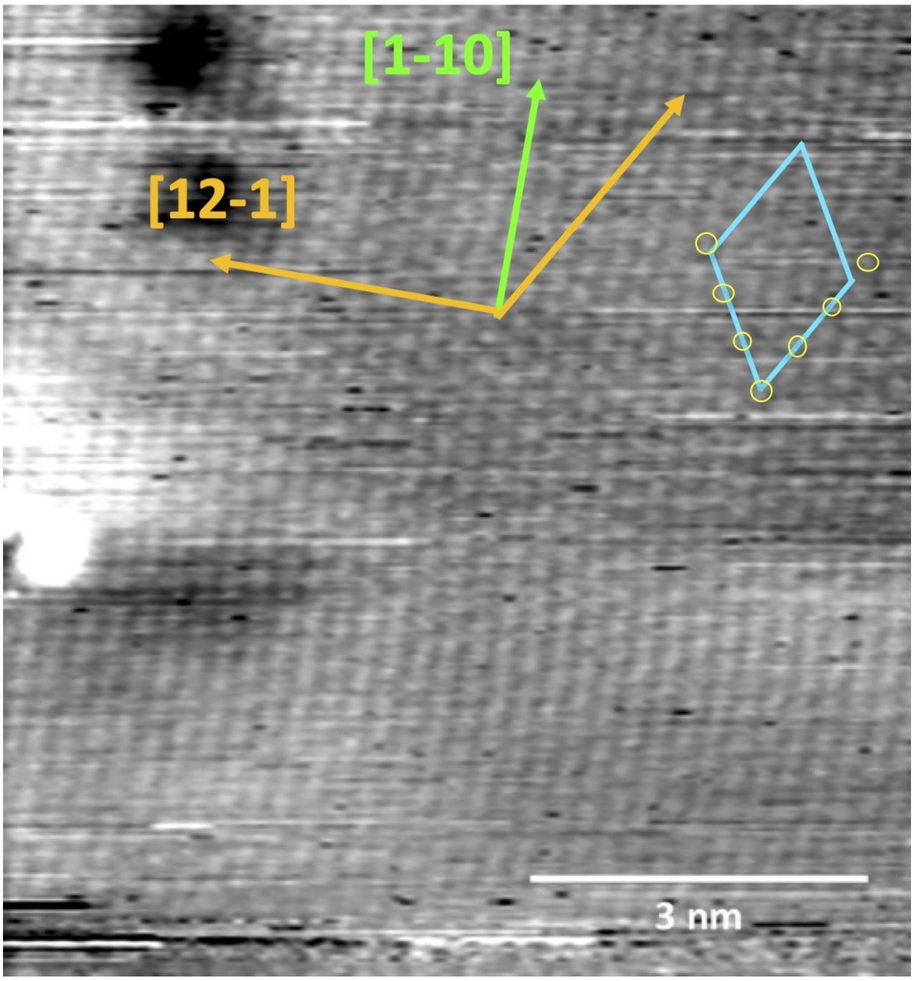

Figure S12: STM images were calibrated to reference topographic images of the clean surface by assuming a bulk plane termination with lattice parameter of 2.88 Å. In the upper image (+945 mV, 1480 pA), the main symmetry directions are superimposed to the surface, as well as the superlattice unit cell of the *hex*-phase (blue lines) formed by MIC **1** deposition at RT. For a better visual comparison, we highlighted by yellow circles the position of the Au atoms. Please notice the small deviations of the atomic rows from the symmetry directions due to the local herringbone reconstruction.

References

(1) Eitzinger, A.; Reitz, J.; Antoni, P. W.; Mayr, H.; Ofial, A. R.; Hansmann, M. M. Pushing the Upper Limit of Nucleophilicity Scales by Mesoionic N-Heterocyclic Olefins. *Angew. Chem., Int. Ed.* **2023**, *62* (40), e202309790. https://doi.org/10.1002/anie.202309790.

(2) Crudden, C. M.; Horton, J. H.; Narouz, M. R.; Li, Z.; Smith, C. A.; Munro, K.; Baddeley, C. J.; Larrea, C. R.; Drevniok, B.; Thanabalasingam, B.; McLean, A. B.; Zenkina, O. V.; Ebralidze, I. I.; She, Z.; Kraatz, H.-B.; Mosey, N. J.; Saunders, L. N.; Yagi, A. Simple Direct Formation of Self-Assembled N-Heterocyclic Carbene Monolayers on Gold and Their Application in Biosensing. *Nat. Commun.* **2016**, *7* (1), 12654. https://doi.org/10.1038/ncomms12654.

(3) Solomon, J. L.; Madix, R. J.; Stöhr, J. Orientation and Absolute Coverage of Benzene, Aniline, and Phenol on Ag(110) Determined by NEXAFS and XPS. *Surf. Sci.* **1991**, *255* (1–2), 12–30. https://doi.org/10.1016/0039-6028(91)90008-g.

(4) Giannozzi, P.; Baroni, S.; Bonini, N.; Calandra, M.; Car, R.; Cavazzoni, C.; Ceresoli, D.; Chiarotti, G. L.; Cococcioni, M.; Dabo, I.; et al. QUANTUM ESPRESSO: A Modular and Open-Source Software Project for Quantum of Materials. *J. Phys.: Condens. Matter* **2009**, *21* (39), 395502. https://doi.org/10.1088/0953-8984/21/39/395502.

(5) Giannozzi, P.; Andreussi, O.; Brumme, T.; Bunau, O.; Buongiorno Nardelli, M.; Calandra, M.; Car, R.; Cavazzoni, C.; Ceresoli, D.; Cococcioni, M.; et al. Advanced Capabilities for Materials Modelling with Quantum ESPRESSO. *J. Phys.: Condens. Matter* **2017**, *29* (46), 465901. https://doi.org/10.1088/1361-648x/aa8f79.

(6) Perdew, J. P.; Burke, K.; Ernzerhof, M. Generalized Gradient Approximation Made Simple. *Phys. Rev. Lett.* **1996**, *77* (18), 3865–3868. https://doi.org/10.1103/PhysRevLett.77.3865.

(7) Garrity, K. F.; Bennett, J. W.; Rabe, K. M.; Vanderbilt, D. Pseudopotentials for High-Throughput DFT Calculations. *Comput. Mater. Sci.* **2014**, *81*, 446–452. https://doi.org/10.1016/j.commatsci.2013.08.053.

(8) Ehrlich, S.; Moellmann, J.; Reckien, W.; Bredow, T.; Grimme, S. System-Dependent Dispersion Coefficients for the DFT-D3 Treatment of Adsorption Processes on Ionic

Surfaces. *ChemPhysChem* **2011**, *12* (17), 3414–3420.
https://doi.org/10.1002/cphc.201100521.

(9) Pehlke, E.; Scheffler, M. Evidence for Site-Sensitive Screening of Core Holes at the Si and Ge (001) Surface. *Phys. Rev. Lett.* **1993**, *71* (14), 2338.
https://doi.org/10.1103/PhysRevLett.71.2338.

(10) Bianchettin, L.; Baraldi, A.; De Gironcoli, S.; Lizzit, S.; Petaccia, L.; Vesselli, E.; Comelli, G.; Rosei, R. Geometric and Electronic Structure of the N/Rh(100) System by Core-Level Photoelectron Spectroscopy: Experiment and Theory. *Phys. Rev. B: Condens. Matter Mater. Phys.* **2006**, *74* (4), 045430. https://doi.org/10.1103/PhysRevB.74.045430.

(11) Triguero, L.; Pettersson, L. G. M.; Ågren, H. Calculations of Near-Edge X-Ray-Absorption Spectra of Gas-Phase and Chemisorbed Molecules by Means of Density-Functional and Transition-Potential Theory. *Phys. Rev. B* **1998**, *58* (12), 8097–8110.
https://doi.org/10.1103/PhysRevB.58.8097.

(12) Leetmaa, M.; Ljungberg, M. P.; Lyubartsev, A.; Nilsson, A.; Pettersson, L. G. M. Theoretical Approximations to X-Ray Absorption Spectroscopy of Liquid Water and Ice. *J. Electron Spectrosc. Relat. Phenom.* **2010**, *177* (2–3), 135–157.
https://doi.org/10.1016/j.elspec.2010.02.004.

(13) Gougoussis, C.; Calandra, M.; Seitsonen, A. P.; Mauri, F. First-Principles Calculations of X-Ray Absorption in a Scheme Based on Ultrasoft Pseudopotentials: From α-Quartz to High-Tc Compounds. *Phys. Rev. B: Condens. Matter Mater. Phys.* **2009**, *80* (7), 075102.
https://doi.org/10.1103/PhysRevB.80.075102.

(14) Fratesi, G.; Lanzilotto, V.; Stranges, S.; Alagia, M.; Brivio, G. P.; Floreano, L. High Resolution NEXAFS of Perylene and PTCDI: A Surface Science Approach to Molecular Orbital Analysis. *Phys. Chem. Chem. Phys.* **2014**, *16* (28), 14834–14844.
https://doi.org/10.1039/c4cp01625d.

(15) Fratesi, G.; Lanzilotto, V.; Floreano, L.; Brivio, G. P. Azimuthal Dichroism in Near-Edge X-Ray Absorption Fine Structure Spectra of Planar Molecules. *J. Phys. Chem. C* **2013**, *117* (13), 6632–6638. https://doi.org/10.1021/jp312569q.

(16) Baby, A.; Lin, H.; Brivio, G. P.; Floreano, L.; Fratesi, G. Core-Level Spectra and Molecular Deformation in Adsorption: V-Shaped Pentacene on Al(001). *Beilstein J. Nanotechnol.* **2015**, *6*, 2242–2251. https://doi.org/10.3762/bjnano.6.230.

(17) Grimme, S.; Bannwarth, C.; Shushkov, P. A Robust and Accurate Tight-Binding Quantum Chemical Method for Structures, Vibrational Frequencies, and Noncovalent Interactions of Large Molecular Systems Parametrized for All spd-Block Elements (Z = 1–86). *J. Chem. Theory Comput.* **2017**, *13* (5), 1989–2009. https://doi.org/10.1021/acs.jctc.7b00118.

(18) Kühne, T. D.; Iannuzzi, M.; Del Ben, M.; Rybkin, V. V.; Seewald, P.; Stein, F.; Laino, T.; Khaliullin, R. Z.; Schütt, O.; Schiffmann, F.; et al. CP2K: An Electronic Structure and Molecular Dynamics Software Package—Quickstep: Efficient and Accurate Electronic Structure Calculations. *J. Chem. Phys.* **2020**, *152* (19), 194103. https://doi.org/10.1063/5.0007045.
